# Differential expression of POMC-processing genes in corticotroph tumors

**DOI:** 10.1530/EO-26-0003

**Published:** 2026-04-21

**Authors:** Elisa Lamback, Renan L Miranda, Laryssa Mendonça, Camila S de Figueiredo, Kaio C R Salum, Rômulo S Dezonne, Luiz Eduardo Wildemberg, Mônica R Gadelha

**Affiliations:** ^1^ Neuroendocrinology Research Center, Endocrinology Section, Medical School and Hospital Universitário Clementino Fraga Filho, Universidade Federal do Rio de Janeiro, Rio de Janeiro, Brazil; ^2^ Neuropathology and Molecular Genetics Laboratory, Instituto Estadual do Cérebro Paulo Niemeyer, Secretaria Estadual de Saúde, Rio de Janeiro, Brazil; ^3^ Neuroendocrine Unit, Instituto Estadual do Cérebro Paulo Niemeyer, Secretaria Estadual de Saúde, Rio de Janeiro, Brazil; ^4^ Laboratory of Biotechnology, Bioengineering and Nanostructured Biomaterials, Institute of Biomedical Science, Universidade Federal do Rio de Janeiro, Rio de Janeiro, Brazil

**Keywords:** corticotroph tumor, Cushing disease, proopiomelanocortin, *USP8*, *TBX19*, *PCSK1*

## Abstract

**Objective:**

Corticotroph tumors (CTs) derive from the *TBX19* lineage and are functioning (FCTs) or nonfunctioning (NFCTs). In FCTs, the main pathogenic variants are found in *USP8,* enhancing proopiomelanocortin (*POMC*) transcription through epidermal growth factor receptor (EGFR) signaling, resulting in a higher secretion index compared with wild type (WT). POMC is cleaved by prohormone convertase 1/3, encoded by proprotein convertase subtilisin/kexin type 1 gene (*PCSK1*), into ACTH. *PCSK1* is inhibited by *PCSK1N,* which in turn is inhibited by transcription factor paired box 6 (*PAX6*). We aimed to compare gene expressions involved in POMC processing among NFCTs, *USP8*+ FCTs, and WT FCTs.

**Methods:**

Fresh CTs were collected to quantify *TBX19*, *POMC*, *EGFR*, *PCSK1*, *PCSK1N,* and *PAX6* by polymerase chain reaction. Sanger sequencing was performed to detect *USP8* variants. ACTH levels were normalized to tumor diameter to calculate the secretion index.

**Results:**

We included 42 NFCTs, 13 WT FCTs, and 11 *USP8*+ FCTs. NFCTs had lower *TBX19*, *POMC*, and *PAX6* compared with both FCT groups, but similar *PCSK1N*. *TBX19* correlated positively with *POMC* (*R* = +0.460; *P* = 0.002) and *PAX6* (*R* = +0.327; *P* = 0.030). *USP8+* FCTs had a higher secretion index (*P* = 0.019), higher *PCSK1* (*P* = 0.037), and also lower *PCSK1N* (*P* = 0.041), compared with WT, despite similar *TBX19* and *POMC*. Secretion index only correlated with *PCSK1N* (*R* = −0.469; *P* = 0.021).

**Conclusions:**

In NFCTs, low *TBX19* may contribute to their nonfunctioning phenotype. In FCTs, *USP8*+ and WT displayed similar *POMC* levels, but downstream *POMC*, *USP8*+ had a higher *PCSK1* and lower *PCSK1N*, which may account for their comparatively increased secretory activity.

## Introduction

Pituitary tumors are classified according to the last World Health Organization classification based on lineage-specific pituitary transcription factors (1). Corticotroph tumors (CTs) derive from T-box family member TBX19 (TPIT) lineage (1). They can be functioning (FCTs), leading to a clinical syndrome known as Cushing’s disease (CD) caused by hypersecretion of adrenocorticotrophic hormone (ACTH), or clinically nonfunctioning tumors (NFCTs) (2). CD is a life-threatening condition caused by hypercortisolism that has negative effects on multiple organs, leading to cardiovascular complications, osteoporosis, diabetes mellitus, thrombosis, cognitive and psychiatric dysfunctions, immune suppression, and increased mortality (2, 3). The main treatment is surgical resection with remission achieved in 81% of CD patients at our center, consistent with the literature; however, 27% of patients experienced recurrence during long-term follow-up (4, 5). In addition, pituitary-directed medications available for CD are cabergoline and pasireotide; however, they show limited efficacy for CD. Cabergoline, which is a DRD2-selective agonist, demonstrates an initial efficacy of approximately 40%, which decreases to 20–25% over time due to escape, and is more effective in combined therapies (6). Pasireotide, which preferentially binds to the somatostatin receptor type 5 (SST5), shows an efficacy of about 15–40% (6, 7). Other treatment options include the USP8 inhibitor RA-9 and steroidogenic inhibitors. Preclinical studies show that they demonstrate high efficacy but may be associated with adverse effects that restrict broader clinical application (2, 8, 9). Therefore, the development of novel drugs for CD is desirable (10).

On the other hand, NFCTs are silent pituitary tumors of corticotrophic lineage, diagnosed when they are large enough to compress local structures or are discovered incidentally (11). Several theories have been proposed; the currently most accepted hypothesis is that they may result from defective processing of proopiomelanocortin (POMC) (12). POMC is cleaved into ACTH by prohormone convertase PC1/3 (encoded by proprotein convertase subtilisin/kexin type 1 gene, *PCSK1*) that is regulated by its inhibitor, PCSK1N or ProSAAS, encoded by *PCSK1N* (13, 14). *PCSK1N* is inhibited by transcription factor paired box 6 (*PAX6)* in mice and pancreatic cells, and *PAX6* has recently been identified in human CTs (15, 16, 17). Upstream of *POMC* transcription is the T-box transcription factor 19 gene (*TBX19*), which encodes the transcription factor TPIT of the corticotroph lineage. TPIT activates *POMC* and is required for cell-specific expression of the *POMC* gene (14, 18). The inactivation of *TBX19* results in loss of *POMC* expression in corticotroph cells (14, 18).

Genetically, the most common somatic pathogenic variants in FCTs are found in the *USP8* gene, with an estimated prevalence of 31%, varying from 0 to 67% according to a recent meta-analysis that analyzed 2,171 cases (19, 20). Pathogenic variants in this gene confer a specific tumor phenotype characterized by smaller size, lower invasiveness, and higher secretion index and remission rates seen in patients, and higher ACTH release and better responsiveness to pasireotide in cell culture, compared to those harboring wild-type (WT) *USP8* tumors (19, 20, 21, 22, 23, 24). FCTs can also harbor pathogenic variants in the *USP48* and *BRAF* genes in approximately 13 and 7% of cases, respectively (2, 25, 26, 27, 28, 29). Somatic pathogenic variants in *USP8* can also be found in up to 10% of NFCTs (30, 31, 32). No pathogenic variants in *USP48* or *BRAF* have been identified in NFCTs.

Hotspot *USP8* activating variants were described, *in vitro*, to decrease epidermal growth factor receptor (EGFR) degradation and increase its recycling, thereby upregulating *POMC* transcription and increasing plasma ACTH concentrations (33).

We aimed to compare the expressions of POMC-processing genes across corticotroph tumor subtypes – NFCTs, *USP8*–WT FCTs, and *USP8*+ FCTs with high secretory activity – to elucidate mechanisms underlying their functional spectrum.

## Materials and methods

### Patients

Ethical approval was obtained from local institutional review board (registered at Plataforma Brasil: CAAE 24075019.5.00000.8110), with consent form collected from patients. Patients were selected from a cohort of treatment-naïve patients with CD and nonfunctioning pituitary tumors (NFPTs) operated at Instituto Estadual do Cérebro Paulo Niemeyer from August 2013 to October 2024. Patients were included if fresh-frozen tumor tissue was available for analysis and histopathology confirmed a corticotroph tumor. Clinical, biochemical, radiological, and histopathological data were collected retrospectively from medical files.

CD was investigated in patients with clinical signs and symptoms of hypercortisolemia and diagnosed as previously described (4). ACTH was measured using the Immulite 2000 assay (Research Resource Identifier (RRID): AB_2783635, polyclonal antibody). Serum cortisol was assessed with the Elecsys Cortisol II kit (RRID: AB_2802131, monoclonal antibody). Urinary free cortisol (UFC) and late-night salivary cortisol (LNSC) assays changed over the years, so we calculated how many times UFC and LNSC were compared to the upper limit of normal range (xULN). In FCTs, tumor secretion index was calculated by dividing ACTH levels by the largest tumor diameter (21).

Patients with NFCTs were identified from a cohort of NFPTs who do not have clinical features of hypercortisolism and were diagnosed due to compressive symptoms or discovered incidentally, and confirmed in histopathological assessment (11).

### Imaging and histopathological studies

The largest tumor diameter was assessed with a magnetic resonance imaging (MRI) scan performed in a 1.5 T GE 450w Optima scanner, with gadolinium contrast.

Histopathological diagnosis included histological and immunohistochemical studies. For light microscopy, 3 μm sections of formalin-fixed and paraffin-embedded tissue were stained with hematoxylin and eosin. Immunohistochemistry was performed to demonstrate the presence of ACTH and/or TPIT, and Ki-67, as previously published (4). The absolute number of mitosis was counted in 10 high-power fields (HPFs) in hematoxylin and eosin slides. Somatostatin receptor type 5 (SST5) (RRID:AB_10859946, monoclonal antibody) was analyzed using the immunoreactive score (IRS), with ≥6 considered as a high score (34). In the original study by Gatto *et al.*, they assessed the expression of somatostatin receptor type 2 (34), and we generalized the score to SST5.

### DNA and RNA extractions

During surgery, tumor samples were collected in saline solution and separated macroscopically from nontumorous material. Samples were immediately frozen at −80°C until DNA and RNA extractions. Total RNA and DNA were extracted from 20 µg of tumor fragment with AllPrep DNA/RNA/miRNA Universal Kit^®^ (Qiagen, Germany). Spectrophotometric reading at NanoDrop One (Thermo Fisher, USA) at a wavelength of 260 nm was used for quantification. RNA was considered pure by calculating the optic density ratio at 260 and 280 nm with an optimum ratio of 1.9–2.0. To eliminate genomic DNA contamination, RNA was treated with DNase Turbo DNA-free^TM^ kit (Invitrogen, USA) and subsequently quantified by fluorescence using the Qubit 3.0 fluorometer (Invitrogen).

### Gene expression analysis

SuperScript® IV First-Strand Synthesis System (Invitrogen) was used for reverse transcription according to the manufacturer’s protocol to produce cDNA at a concentration of 50 ng/μL. We assessed *EGFR* (Taqman assay Hs01076088_m1), *TBX19* (Hs00193027_m1), *POMC* (Hs00174947_m1), *PCSK1* (Hs00175619_m1), *PCSK1N* (Hs00560041_m1), and *PAX6* (Hs01088114_m1) mRNA expressions by digital droplet polymerase chain reaction (ddPCR) in available fresh samples using glyceraldehyde 3-phosphate dehydrogenase (*GAPDH*; Hs99999905_m1) as a reference gene and reported them in relative expression corrected by *GAPDH* expression. This endogenous control gene was chosen based on the selection and validation of reliable reference genes in pituitary tumors (35).

Since most FCTs are microadenomas (<1 cm in diameter) and the available fresh-frozen specimens were often even smaller, we employed ddPCR, a high-precision method that performs reliably with low template abundance, enabling robust quantification from minimal cDNA amount. PCRs were conducted in duplicate for each sample with a 20 µL reaction mixture that consisted of 10 µL of ddPCR supermix (Bio-Rad, USA), 1 µL of Taqman assay, 8 µL of nuclease-free water, and 1 µL of cDNA. Five nanograms of cDNA were used for genes; however, *POMC*, *TBX19*, and *PCSK1N* were highly expressed. Therefore, to avoid saturation of droplets, we diluted the samples 1:10 using 0.5 ng of cDNA for these three specific genes. *GAPDH* expression was evaluated with 0.5 and 5 ng to properly adjust expression levels. Droplets were generated with the QX100 Droplet Generator (Bio-Rad) and amplified and read on QX200 Droplet Reader (Bio-Rad). Thermocycling conditions were 95°C for 10 min and 35 successive cycles at 95°C for 15 s and 60°C for 1 min.

In addition, to exclude normal pituitary tissue contamination in FCTs, which are usually small tumors, quantitative reverse transcription polymerase chain reaction (qRT-PCR) of *PIT1* (a transcription factor of mammosomatotroph lineage; Hs01030492_m1) was performed in FCT samples and compared with three normal pituitary glands obtained from cadavers with no pituitary disease (post mortem interval of <12 h). Tumors expressing >8% of *PIT1* mRNA relative to the mean normal pituitary samples were excluded, as suggested by Hayashi (36).

### *USP8*, *USP48*, and *BRAFV600E* sequencing

Direct Sanger sequencing was performed to detect pathogenic variants in *USP8* exon 14 in both FCTs and NFCTs, as previously published (37). In FCTs, *USP48* exon 10 and *BRAF* exon 15 were also sequenced to identify and exclude tumors harboring variants in these genes. Wild-type FCTs were defined as those lacking pathogenic variants in *USP8*, *USP48*, or *BRAF*. The primer pair used for *USP8* sequencing generated a 235bp amplicon (forward primer: 5′-CTT​GAC​CCA​ATC​ACT​GGA​AC-3′; reverse primer: 5′-TTA​CTG​TTG​GCT​TCC​TCT​TCT​C-3′). For *USP48,* a 502bp amplicon was obtained (forward primer: 5′-TGTGCCCGGCTTCAGTTA-3′; reverse primer: 5′-ACA​TTT​GCC​TGC​TAT​AAT​CCT​GG-3′). For *BRAF,* a 405bp amplicon was produced (forward primer: 5′-TCC​TAA​CAC​ATT​TCA​AGC​CCC​A-3′; reverse primer: 5′-CAG​CAT​CTC​AGG​GCC​AAA​AAT-3′).

Regarding *USP8* variants*,* the nomenclature p.S719del is interchangeable with p.S718del, as these codons represent identical shortening of two consecutive serine amino acids to one. We used codon 719, according to the recommended nomenclature of the Human Genome Variation Society (38).

### Statistical analysis

Categorical variables are expressed as percentages, and comparisons between groups were performed using chi-square test or Fisher’s exact test, as appropriate. Continuous variables were expressed as median (minimum–maximum). Comparisons among the three groups were performed with the Kruskal–Wallis test; when significant (*P* < 0.05), pairwise comparisons were conducted with the Mann–Whitney test with Bonferroni correction. Spearman’s rank correlation was used to examine associations between continuous variables.

### External database analysis

To compare our data with an independent cohort, we analyzed the RNA-seq data of CTs from the pangenomic classification published by Neou *et al.* (database E-MTAB-7768 available at the European Bioinformatics Institute Array Express) (22). This database has RNA-seq data from 135 pituitary tumors, of which 35 are of corticotrophic origin. We removed eight samples (1 Nelson’s and 7 subclinical CD), resulting in a total of 19 FCT (10 *USP8*+ and 9 WT) and 8 NFCT samples. The normalization and differential gene expression were performed with DESeq2 (39). We considered genes differentially expressed that exhibited a Log^2^ fold change >1 or <−1 and an adjusted *P*-value of 0.01.

## Results

### Description of the corticotroph tumor cohorts

A total of 71 tumors were initially selected: 44 NFCTs and 27 FCTs. Pathogenic variants in *USP8* were found in two NFCTs (2/44, 4.5%), both p.S719P; these cases were excluded from group comparisons due to the small sample size. In addition, of the 27 FCTs, two were excluded because they harbored the *USP48* p.M415I pathogenic variant, and one was excluded due to >8% *PIT1* mRNA expression relative to the mean of three normal pituitary samples. No *BRAFV600E* variants were identified. Therefore, 66 tumors were included: 42 NFCTs and 24 FCTs, of which 11 (45.8%) were *USP8*+ and 13 (54.2%) were WT.

**Figure 1:**
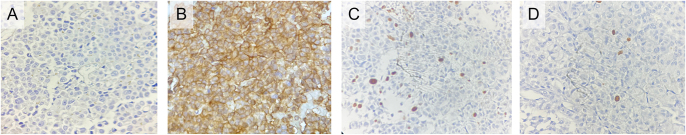
Ki-67 and somatostatin receptor type 5 immunostaining (magnification 40×). (A) Somatostatin receptor type 5 immunoreactive score of 0, (B) somatostatin receptor type 5 immunoreactive score of 12, (C) Ki-67 of 11%, and (D) Ki-67 of 2%.

In the NFCT cohort, the majority of patients were female (28/42; 66.7%) (Table 1 and Supplementary Table 1; see section on Supplementary materials given at the end of the article). At diagnosis, the median age was 52 years (range, 25–73), and the median largest tumor diameter was 3.6 cm (range, 2.0–8.2). Histopathological analysis revealed a median Ki-67 of 2.0% (range, 0.2–11.0), a median mitotic count of 0 (range, 0–7), and a median SST5 IRS of 0 (range, 0–6) (Fig. 1). Thirty-eight tumors were positive for ACTH on immunohistochemistry, and four were TPIT-positive only.

**Table 1:** Patient and tumor characteristics among NFCTs, *USP8*+ FCTs, and WT FCTs.

Characteristics	NFCT (*n* = 42)	WT FCT (*n* = 13)	*USP8+* FCT (*n* = 11)	*P*-value	*P*-value NFCT × WT FCT	*P*-value NFCT × *USP8*+ FCT	*P*-value *USP8*+ FCT × WT FCT
Age (years)	52 (25–73)	37 (20–60)	37 (23–52)	**<0.001**	**0.009**	**0.004**	1.000
Female gender (%)	28 (66.7%)	10 (76.9%)	11 (100%)	0.086	-	-	-
Cortisol post-DST (mcg/dL) Cortisol post-DST (nmol/L)	NA	21.0 (8.6–32.0) 579.3 (237.2–882.8), *n* = 7	24.3 (4.3–48.7) 670.3 (118.6–1,343.3), *n* = 7	-	-	-	0.902
LNSC (xULN)	NA	3.0 (1.9–6.4), *n* = 9	3.3 (1.6–44.2), *n* = 10	-	-	-	0.814
UFC (xULN)	NA	2.5 (1.9–12.5), *n* = 9	4.7 (1.1–13.9), *n* = 6	-	-	-	0.380
ACTH (pg/mL) ACTH (pmol/L)	NA	72.0 (28.7–224.0) 15.6 (6.3–49.3)	65.0 (41.0–330.0) 14.3 (9.0–72.7)	-	-	-	0.733
Largest tumor diameter (cm)	3.6 (2.0–8.2)	1.9 (0.6–4.3)	1.0 (0.4–1.6)	**<0.001**	**0.001**	**0.001**	0.199
Secretion index	NA	47.8 (16.4–86.2)	67.1 (46.9–235.7)	-	-	-	**0.019**
Ki-67 (%)	2.0 (0.2–11.0)	1.8 (0.1–4.7)	2.0 (0.2–11.5)	0.588	-	-	-
Mitoses (per 10 HPFs)	0 (0–7)	0 (0–2)	0 (0–4)	0.641	-	-	-
Median SST5 IRS	0 (0–6), *n* = 13	1 (0–12)	12 (1–12)	**<0.001**	0.762	**<0.001**	**0.015**

Bold indicates statistical significance. NFCT, nonfunctioning corticotroph tumor; FCT, functioning corticotroph tumor; USP8+, tumor with a USP8 pathogenic variant; WT, wild type; ACTH, adrenocorticotrophic hormone; LNSC, late night salivary cortisol; ULN, upper limit of normal; UFC, urinary free cortisol; DST, dexamethasone suppression test; HPF, high-power field; SST5, somatostatin receptor type 5; and IRS, immunoreactive score.

In the *USP8*+ FCT cohort, all patients were female (11/11; 100%) (Table 1). At diagnosis, the median age was 37 years (range, 23–52). Median biochemical values were as follows: cortisol post-dexamethasone suppression test (DST) 24.3 μg/dL (range, 4.3–48.7) (670.3 nmol/L (range, 118.6–1,343.3)); LNSC 3.3 xULN (range, 1.6–44.2); UFC 4.7 xULN (range, 1.1–13.9); and ACTH 65.0 pg/mL (range, 41.0–330.0) (14.3 pmol/L (range, 9.0–72.7)). The median largest tumor diameter was 1.0 cm (range, 0.4–1.6), and the median secretion index was 67.1 (range, 46.9–235.7). Histopathological analysis revealed a median Ki-67 index of 2.0% (range, 0.2–11.5), a median mitotic count of 0 (range, 0–4.0), and a median SST5 IRS of 12 (range, 1–12). Pathogenic *USP8* variants included p.P720R (*n* = 7), p.S719del (*n* = 3), and p.S719P (*n* = 1).

In the WT FCT cohort, the majority of patients were female (10/13; 76.9%) (Table 1). At diagnosis, the median age was 37 (range, 20–60). Median biochemical values were as follows: cortisol post-dexamethasone suppression test (DST) 21.0 μg/dL (range, 8.6–32.0) (579.3 nmol/L (range, 237.2–882.8)); LNSC 3.0 xULN (range, 1.9–6.4); UFC 2.5 xULN (range, 1.9–12.5); and ACTH 72.0 pg/mL (range, 28.7–224.0) (15.6 pmol/L (range, 6.3–49.3)). The median largest tumor diameter was 1.9 cm (range, 0.6–4.3), and the median secretion index was 47.8 (range, 16.4–86.2). Histopathological analysis revealed a median Ki-67 index of 1.8% (range, 0.1–4.7), a median mitotic count of 0 (range, 0–2.0), and a median SST5 IRS of 1 (range, 0–12).

### Comparison of the corticotroph tumor cohorts

NFCT patients were older than *USP8*+ FCT patients (median age 52 years (25–73) vs 37 (23–52); *P* = 0.004) and WT FCT patients (37 (range, 20–60); *P* = 0.009). NFCTs were larger than *USP8*+ FCTs (median 3.6 cm (range, 2.0–8.2) vs 1.0 (range, 0.4–1.6); *P* < 0.001) and WT FCTs (1.9 cm (range, 0.6–4.3); *P* = 0.001). NFCTs also had a lower SST5 IRS than *USP8*+ FCTs (0 (range, 0–6) vs 12 (range, 1–12); *P* < 0.001). In addition, *USP8*+ FCTs had a higher SST5 IRS compared to WT FCTs (12 (range, 1–12) vs 1 (range, 0–12); *P* = 0.015). Despite similar ACTH concentrations and tumor diameters, *USP8+* FCTs exhibited a higher secretion index than WT FCTs (67.1 (range, 46.9–235.7) vs 47.8 (range, 16.4–86.2); *P* = 0.019) (Table 1 and Supplementary Table 1).

No difference was seen between groups regarding sex, biochemical assessments, Ki-67 proliferation index, and mitosis.

### Differential expression of genes involved in POMC processing in CTs

**Figure 2:**
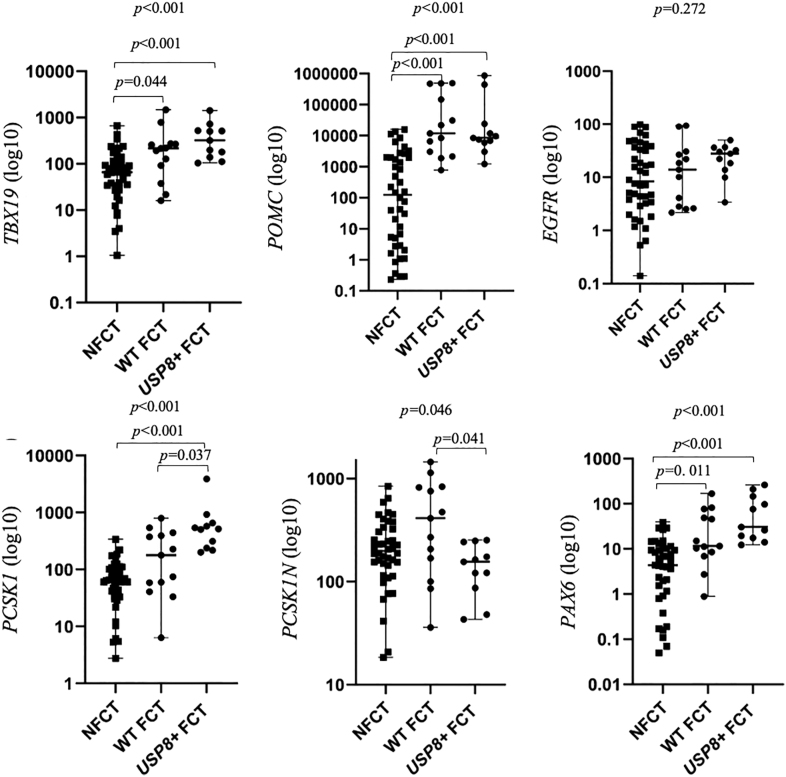
Gene expression in corticotroph tumors. FCT, functioning corticotroph tumor; *USP8*+, tumor with a *USP8* pathogenic variant; WT, wild type; NFCT, nonfunctioning corticotroph tumor; *TBX19*, T-box family member 19; *POMC*, proopiomelanocortin; *EGFR*, epidermal growth factor receptor; *PCSK1*, proprotein convertase subtilisin/kexin type 1; *PCSK1N*, inhibitor of *PCSK1*; and *PAX6*: paired box 6.

NFCTs had lower expression of *TBX19*, *POMC*, *PCSK1*, and *PAX6* than *USP8*+ FCTs (all *P* < 0.001) and lower *TBX19* (*P* = 0.044), *POMC* (*P* < 0.001), and *PAX6* (*P* = 0.011) than WT FCTs. Although *POMC* and *PCSK1* were lower in NFCTs, the expression of *PCSK1N* was similar to that in both FCT groups (Table 2; Fig. 2). *PCSK1/PCSK1N* ratio was 0.3 (range, 0.0–2.5) in NFCTs, 0.8 (range, 0.0–2.3) in WT FCTs, and 2.8 (range, 0.9–81.5) in *USP8*+ FCTs.

**Table 2:** Gene expression in NFCTs, *USP8*+ FCTs, and WT FCTs.

Characteristics	NFCT (*n* = 42)	WT FCT (*n* = 13)	*USP8+* FCT (*n* = 11)	*P*-value	*P*-value NFCT × WT FCT	*P*-value NFCT × *USP8*+ FCT	*P*-value *USP8*+ FCT × WT FCT
*TBX19*	65.9 (1.1–665.2)	216.0 (16.1–1,472.4)	319.9 (104.8–1,416.9)	**<0.001**	**0.044**	**<0.001**	0.615
*POMC*	123.4 (0.2–15,891.4)	11,773.6 (770.0–494,813.2)	8,392.1 (1,227.0–863,086.4)	**<0.001**	**<0.001**	**<0.001**	1.000
*EGFR*	8.3 (0.1–99.1)	13.9 (2.2–94.1)	28.2 (3.4–50.1)	0.272	**-**	**-**	-
*PCSK1*	63.7 (2.8–339.2)	177.9 (6.4–799.1)	519.3 (202.1–3,900.4)	**<0.001**	0.129	**<0.001**	**0.037**
*PCSK1N*	197.6 (18.5–840.2)	411.9 (36.0–1,448.5)	155.6 (43.1–253.1)	**0.046**	0.258	0.509	**0.041**
*PAX6*	4.3 (0.0–39.4)	11.6 (0.9–168.1)	27.9 (12.3–263.2)	**<0.001**	**0.011**	**<0.001**	0.368

Bold indicates statistical significance. NFCT, nonfunctioning corticotroph tumor; FCT, functioning corticotroph tumor; *USP8*+, tumor with a *USP8* pathogenic variant; WT, wild type; *TBX19*, T-box family member 19; *POMC*, proopiomelanocortin; *EGFR*, epidermal growth factor receptor; *PCSK1*, proprotein convertase subtilisin/kexin type 1; *PCSK1N*, inhibitor of *PCSK1*; and *PAX6*, paired box 6.

In NFCTs, *TBX19* correlated positively with *POMC* (*R* = +0.460; *P* = 0.002) and *PAX6* (*R* = +0.327, *P* = 0.030), but not with *PCSK1* (*P* = 0.179).

*USP8*+ and WT FCTs had similar *TBX19* (*P* = 0.615) and *POMC* (*P* = 1.000) expressions, with no correlation between these variables (*P* = 0.253) in the FCT groups. Downstream *POMC*, *USP8*+ FCTs had higher *PCSK1* (*P* = 0.037) and lower *PCSK1N* (*P* = 0.041) compared to WT FCTs (Table 2; Fig. 2). *PCSK1N* was not correlated with *PCSK1* (*P* = 0.632).

**Figure 3:**
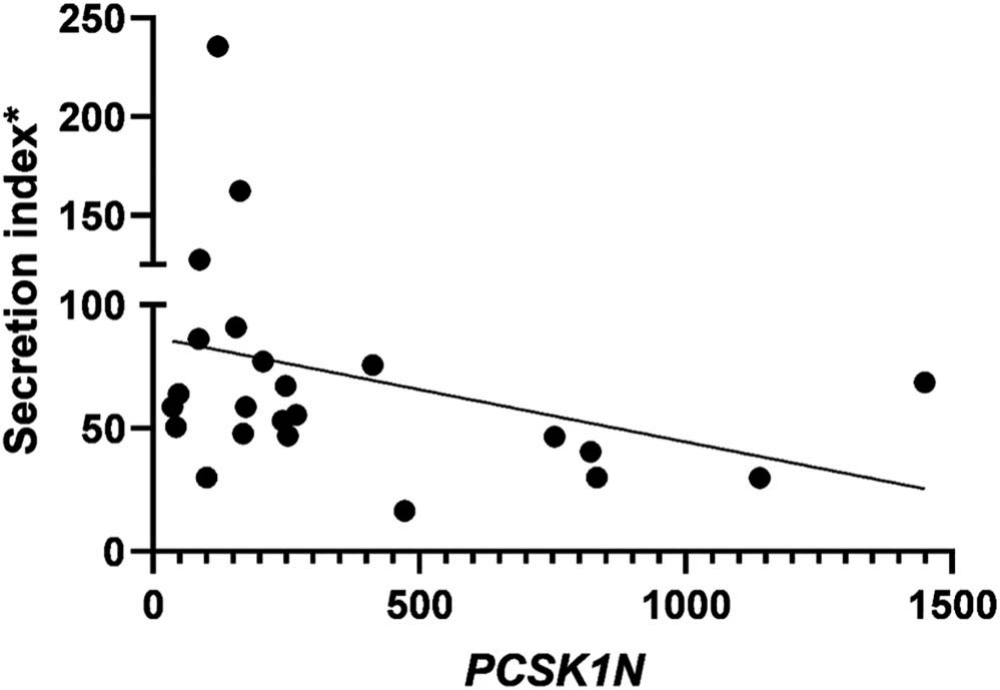
Secretion index and *PCSK1N* expression in functioning corticotroph tumors *PCSK1N*: inhibitor of *PCSK1* (proprotein convertase subtilisin/kexin type 1). *Secretion index: ACTH concentration divided by the largest tumor diameter.

Secretion index only correlated with *PCSK1N* (*R* = −0.469; *P* = 0.021) (Fig. 3). *PCSK1N* was not correlated with any other variables, including tumor diameter (*P* = 0.462), ACTH concentrations (*P* = 0.191), and *PCSK1* (*P* = 0.632). Across all FCTs combined, ACTH levels correlated positively with tumor diameter (*R* = +0.473; *P* = 0.013), and this association remained significant in the WT cohort (*R* = +0.663; *P* = 0.014), but not in the *USP8*+ cohort (*P* = 0.065).

In the entire cohort, *PCSK1* and *POMC* were negatively correlated with age (*R* = −0.335, *P* = 0.006 and *R* = −0.398, *P* < 0.001, respectively) and largest tumor diameter (*R* = −0.531, *P* < 0.001 and *R* = −0.503, *P* < 0.001, respectively) (Supplementary Table 2). *EGFR* expression did not differ among groups.

**Figure 4:**
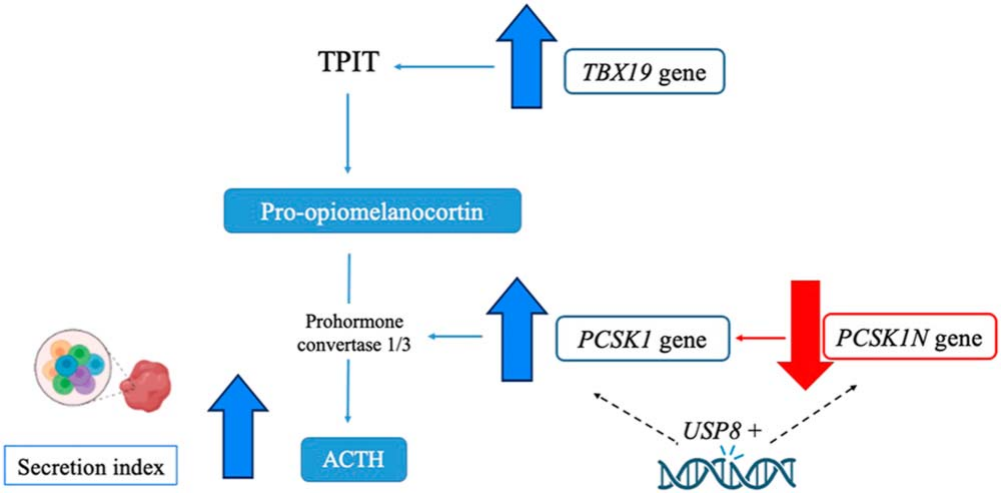
Summary of findings comparing functioning corticotroph tumors to nonfunctioning corticotroph tumors. Genes are highlighted in italic. The blue lines indicate stimulation, the red lines inhibition, and the dotted lines indirect mechanism. By comparing the three groups of corticotroph tumors by secretory behavior (*USP8*+ FCTs with the highest secretion index, followed by WT FCTs with a lower secretion index and NFCTs), we found that *TBX19* and *PCSK1* expressions are increased in *USP8*+ and WT FCTs compared to NFCTs. We also found that, in FCTs, *PCSK1N* expression was decreased in *USP8*+ FCTs compared to WT FCTs. These data suggest that USP8 might act indirectly in *PCSK1* and *PCSK1N* expressions modulating the tumor’s secretion. *TBX19*, T-box family member 19; POMC, proopiomelanocortin; *PCSK1*, proprotein convertase subtilisin/kexin type 1; *PCSK1N*, inhibitor of *PCSK1*; ACTH, adrenocorticotrophic hormone; and *USP8*+, tumor with a *USP8* pathogenic variant.

A figure summarizing overall trends is depicted in Fig. 4.

### External database analysis

Reanalyzing the database E-MTAB-7768, we found lower *TBX19* (*P* = 0.013), lower *PAX6* (*P* = 0.035), and higher *PCSK1N* (*P* = 0.034) in NFCTs compared to FCTs (22), but no difference in *POMC* (*P* = 0.211), *PCSK1* (*P* = 0.158), or *EGFR* (*P* = 0.866) levels. In addition, taking only into account FCTs, *USP8*+ FCTs showed higher *TBX19* (*P* = 0.011) and lower *PCSK1N* (*P* = 0.003) compared to WT FCTs, while no difference was seen in *POMC* (*P* = 0.265), *PCSK1* (*P* = 0.875), *PAX6* (*P* = 0.933), or *EGFR* (*P* = 0.854) (22).

## Discussion

In this study, tumors were characterized by comparing their functional status and the expression of key genes involved in tumor identity and *POMC* processing, including *PCSK1*, *PCSK1N*, *PAX6*, *TBX19*, and *EGFR*, by comparing three groups of secretory behavior. In addition, we assessed the expression levels of two genes not routinely investigated: *PCSK1N* and *PAX6*, with the first one being correlated with the secretion index and the latter detected in CTs. We found that NFCTs had lower *TBX19*, which correlated positively with *POMC* and *PAX6,* while *PCSK1N* levels were similar to those in FCTs. In addition, *PCSK1* was lower in NFCTs compared with *USP8*+ FCTs. Unexpectedly, *POMC* levels were similar between *USP8*+ and WT FCTs. Nonetheless, downstream *POMC*, *USP8*+ FCTs exhibited higher *PCSK1* and also lower *PCSK1N* than WT FCTs. Among FCTs, the secretion index correlated only with *PCSK1N*.

NFCTs had lower *TBX19* compared with FCTs, which is consistent with previous studies (14, 22, 40, 41), although other studies have described similar levels (42, 43, 44). In both our cohort and that of Raverot *et al.*, *TBX19* correlated positively with *POMC* (43). As *TBX19* is essential for *POMC* transcription and corticotroph differentiation, these findings suggest that NFCTs are poorly differentiated tumors (42, 43). In addition, in our study, although *POMC* and *PCSK1* were lower in NFCTs, the levels of *PCSK1N* were similar to those in FCTs, as depicted in Fig. 2, suggesting that for the same level of *PCSK1N* in FCTs and NFCTs, there would be relatively more *PCSK1N* to inhibit *PCSK1* in the NFCT cohort compared to the FCT cohort (as exemplified by the lower *PCSK1/PCSK1N* ratio in NFCTs), and consequently a relatively lower expression of *PCSK1* and *POMC*. This suggests that there would be relatively less POMC at the protein level to be cleaved in the NFCT group, possibly explaining their nonfunctioning properties.

*PCSK1* was consistently lower in NFCTs than in FCTs across different studies, including comparisons with microFCTs (14, 17, 40, 41, 44). A 2010 study further demonstrated that microFCTs differ from both macroFCTs and NFCTs in clinical, pathological, and *POMC* expression profiles, which may be explained, in our view, by a higher prevalence of *USP8* pathogenic variants in microFCTs, as these tumors are typically smaller. In line with this hypothesis, we also observed lower *PCSK1* in NFCTs when compared specifically with *USP8*+ FCTs (43).

Regarding FCTs, several studies have examined the expression of POMC-processing genes (10, 21, 22, 31, 33, 36, 45, 46, 47, 48, 49, 50, 51). In the seminal study by Reincke *et al.* characterizing *USP8*+ FCTs, *USP8*+ was shown to increase EGFR’s recycling, thereby upregulating *POMC* transcription *in vitro* (33). Additional *in vitro* studies support a direct link between USP8 and EGFR signaling, showing increased *POMC* transcription in AtT-20 cells transfected with *USP8*+ and in primary corticotroph cultures (45, 46, 49). In our study, as well as in other *in vivo* studies and in a primary culture study, *EGFR* expression was similar between *USP8*+ and WT tumors, possibly because *USP8+* increases its recycling rather than its expression (21, 22, 31, 36, 51). However, in the study by Ballmann *et al.*, EGFR immunohistochemistry was negative in 40/42 (95.2%) FCTs, including all *USP8*+ tumors (48). Likewise, Hayashi *et al.* did not observe increased EGFR protein expression or activated EGFR signaling in *USP8+* tumors (36). These observations suggest that, *in vivo*, FCT pathogenesis is more complex and may involve mechanisms other than EGFR signaling. Additional pleiotropic effects of *USP8*+ have been suggested. Bujko *et al.* did not find EGFR-related pathways as significantly enriched among the differently expressed genes in a transcriptomic study comparing *USP8*+ and WT FCTs (31). There is evidence that other proteins associated with tumor development may be targets of USP8 (31).

Although initial cell culture studies suggested increased *POMC* expression in *USP8*-mutant AtT-20 cells and in primary cultures, most *in vivo* studies in human FCTs have not confirmed this (21, 33, 45, 46, 49, 52). One study showed that *POMC* was increased, but four others did not find differences in *POMC* levels between groups, as well as our study (22, 31, 36, 51). Based on data kindly shared by Bujko *et al.*, *POMC* expression did not differ between *USP8*+ (*n* = 11) and WT (*n* = 17) FCTs (31). This could theoretically be due to different variants, as Reincke *et al.* initially described that the pathogenic variants p.S719del, p.P720R, and p.S718P deubiquitinated EGFR more efficiently than WT *USP8*, while *USP8*-p.S718C and p.(L713R; Y717C) had activity equivalent to that of WT in AtT-20 cells (33). However, no evidence for variant-specific effect was found after reviewing the literature, as most tumors harbor p.S719del, p.P720R, and p.S718P pathogenic variants (47, 49).

Therefore, USP8 might have other not-yet-well-characterized substrates beyond EGFR, such as transcription factors involved in *POMC* gene expression that might be deubiquitinated by USP8 and lead to increased ACTH (53). Downstream *POMC*, we found increased *PCSK1* and decreased *PCSK1N* in *USP8*+ FCTs compared with WT, with no correlation between *PCSK1* and *PCSK1N*. As the expression of *PCSK1N* was decreased in FCTs, we sequenced the three exons of *PCSK1N* by Sanger sequencing in 20 available FCT samples (data not shown) and found no pathogenic variant that could explain its lower mRNA expression. Polymorphisms in codons 39, 43, 150, and 183 of exon 2 were found in the majority of samples (15/20) and were not related to the presence of *USP8*+. *PCSK1N* has only been described previously in the pangenomic classification – reanalysis of the E-MTAB-7768 database was consistent with our findings of lower *PCSK1N* in *USP8*+ than in WT (22). In our cohort, the secretion index was only correlated with *PCSK1N,* being higher in tumors that expressed lower *PCSK1N,* possibly explaining the higher secretion index seen in *USP8*+ compared with WT FCTs. Whether *PCSK1* or *PCSK1N* is an indirect target of *USP8* remains to be determined.

We hypothesized that higher *PCSK1* and lower *PCSK1N* in *USP8+* tumors might be due to higher expression of *PAX6,* which is important during pituitary embryogenesis (54). *PAX6* downregulates *PCSK1N*, and mice with inactivating pathogenic variants in *PAX6* (PAX6 deficiency) have low PC1/3, as well as low ACTH concentrations (15, 55). *PCSK1N* has also been described as an endogenous inhibitor of *PCSK1* in AtT-20 cells (40). Although these studies are in mice, *PAX6* has been detected recently in human CTs (17). In our study, *PAX6* was detected in CTs, but its expression did not differ between *USP8*+ and WT FCTs, consistent with our reanalysis of the pangenomic classification dataset (22).

This study has some limitations. Protein expression was not assessed due to a small sample size in FCTs, and no functional studies were done to test our findings, although such investigations are warranted.

In conclusion, differential expression of POMC-processing genes may underlie the secretory behavior of CTs. In NFCTs, compared with FCTs, *TBX19* is lower (and positively correlates with *POMC*), while *PCSK1N* is similar; these features may contribute to their nonfunctioning phenotype. In FCTs, *POMC* levels were similar between *USP8*+ and WT FCTs, suggesting that, *in vivo*, *USP8*+ FCT pathogenesis is more complex and may involve mechanisms other than EGFR signaling. Downstream *POMC*, *USP8*+ exhibit higher *PCSK1* and lower *PCSK1N* compared to WT FCTs, which may account for their comparatively increased secretory activity. *PCSK1N* may be a potential new regulator of the secretory properties of CTs.

## Supplementary materials



## Declaration of interest

MRG is an Associate Editor at *Endocrine Oncology*, but played no role in the journal’s evaluation of the manuscript. EL, LEW, and MRG have received speaker fees from Recordati Rare Diseases. MRG is also an advisory board member for Recordati Rare Diseases and Crinetics Pharmaceutical and is a principal investigator for Crinetics. The other authors have nothing to disclose.

## Funding

This work was supported by Fundação Carlos Chagas Filho de Amparo à Pesquisa do Estado do Rio de Janeiro (Grant no E-26/211.294/2021).

## Author contribution statement

EL conceived the study and prepared the original draft of the manuscript. EL, RLM, LM, CSF, KCRS, and RSD designed the methodology. EL, RLM, and LEW performed formal analysis and investigation. LEW reviewed and edited the manuscript. LEW and MRG supervised the study. All authors approved the final version of the manuscript.
