# Supplementary material for: Differential expression of POMC-processing genes in corticotroph tumors
Source: Endocr Oncol. 2026 Apr 21;6(1):e260003. doi: 10.1530/EO-26-0003 (PMC13130876; doi:10.1530/EO-26-0003)
Supplement: Supplementary file 1 [file supplementary_materials.pdf]

Sup. Table 1 Detailed description of the patients

Type	Age (years)	Sex	Invasion	Largest tumor diameter (cm)	Secretion index	ACTH (pg/mL)	UFC ULN	Cortisol post DST (mcg/dL)	LNSC ULN	SST5 IRS	Ki-67 (%)	Mitosis
NFCT	63	F	No	3.1	NA	NA	NA	NA	NA	0	0.9	0
NFCT	40	F	NA	2.6	NA	NA	NA	NA	NA	NA	3.0	0
NFCT	35	F	No	3.0	NA	NA	NA	NA	NA	NA	2.0	0
NFCT	56	F	Yes	5.5	NA	NA	NA	NA	NA	NA	5.0	0
NFCT	66	F	Yes	3.2	NA	NA	NA	NA	NA	NA	2.0	0
NFCT	43	F	Yes	2.2	NA	NA	NA	NA	NA	NA	3.0	0
NFCT	25	F	No	2.8	NA	NA	NA	NA	NA	NA	5.0	5
NFCT	64	M	NA	3.7	NA	NA	NA	NA	NA	NA	1.0	0
NFCT	29	M	Yes	5.2	NA	NA	NA	NA	NA	NA	3.2	7
NFCT	53	F	No	3.9	NA	NA	NA	NA	NA	NA	3.2	2
NFCT	47	M	No	3.2	NA	NA	NA	NA	NA	NA	2.4	2

NFCT	47	F	Yes	3.6	NA	NA	NA	NA	NA	NA	0.5	3
NFCT	37	F	Yes	2.3	NA	NA	NA	NA	NA	NA	3.6	3
NFCT	54	M	Yes	3.9	NA	NA	NA	NA	NA	NA	9.3	7
NFCT	60	F	Yes	6.4	NA	NA	NA	NA	NA	NA	0.2	2
NFCT	38	M	Yes	5.3	NA	NA	NA	NA	NA	NA	1.1	3
NFCT	40	M	Yes	2.9	25.9	75.2	NA	NA	NA	1	0.7	2
NFCT	46	M	No	3.7	NA	NA	NA	NA	NA	0	0.5	1
NFCT	67	F	Yes	4.9	NA	NA	NA	NA	NA	2	2.0	3
NFCT	34	F	No	2.1	45.7	95.9	NA	NA	NA	NA	11.0	2
NFCT	49	M	Yes	3.4	45.0	153.0	NA	NA	NA	1	1.1	0
NFCT	31	F	Yes	7.1	NA	NA	NA	NA	NA	NA	1.5	2
NFCT	63	M	No	4.3	NA	NA	NA	NA	NA	NA	2.5	0
NFCT	58	F	No	2.7	NA	NA	NA	NA	NA	NA	1.4	2
NFCT	65	F	Yes	2.0	NA	NA	NA	NA	NA	NA	1.5	1

NFCT	56	F	No	2.4	71.3	171.0	NA	NA	NA	NA	3.7	0
NFCT	71	F	Yes	2.7	NA	NA	NA	NA	NA	NA	1.2	1
NFCT	56	F	Yes	6.2	NA	NA	NA	NA	NA	NA	0.5	0
NFCT	59	F	Yes	4.5	1.1	5.0	NA	NA	NA	NA	0.8	0
NFCT	58	F	No	2.4	6.1	14.6	NA	NA	NA	NA	3.3	1
NFCT	46	F	Yes	5.3	NA	NA	NA	NA	NA	0	2.0	2
NFCT	73	F	Yes	3.5	NA	NA	NA	NA	NA	NA	1.2	0
NFCT	41	F	Yes	4.0	NA	NA	NA	NA	NA	NA	2.7	0
NFCT	52	F	Yes	4.0	NA	NA	NA	NA	NA	NA	2.6	0
NFCT	59	F	Yes	3.0	NA	NA	NA	NA	NA	6	1.0	0
NFCT	44	M	Yes	2.1	NA	NA	NA	NA	NA	1	2.0	0
NFCT	52	M	Yes	7.7	NA	NA	NA	NA	NA	0	1.0	0
NFCT	40	M	Yes	8.2	5.9	48.7	NA	NA	NA	0	1.2	0
NFCT	70	F	Yes	2.6	20.0	52.0	NA	NA	NA	0	0.4	0
NFCT	58	M	Yes	4.0	NA	NA	NA	NA	NA	1	2.0	0
NFCT	35	F	No	3.3	12.1	40.0	NA	NA	NA	NA	2.5	0
NFCT	46	M	Yes	4.0	NA	NA	NA	NA	NA	0	0.6	0

WT FCT	60	F	NA	2.0	68.5	137.0	2.55	24.6	NA	4	2.0	1
WT FCT	41	F	NA	1.9	30.1	57.1	2.0	NA	1.9	2	1.6	0
WT FCT	46	F	No	1.2	46.5	55.8	1.9	NA	1.9	12	4.7	0
WT FCT	28	F	No	1.3	30.0	39.0	3.1	17.3	2.0	12	0.7	0
WT FCT	33	F	NA	2.1	29.8	62.6	2.1	10.0	3.0	0	0.2	1
WT FCT	30	M	No	3.5	40.5	141.6	8.0	21.0	1.9	1	3.8	2
WT FCT	32	F	No	4.3	16.4	70.6	NA	NA	NA	0	1.3	1
WT FCT	37	M	Yes	1.7	77.1	131.0	NA	8.6	3.1	0	1.8	2
WT FCT	50	M	No	1.6	75.6	121.0	12.5	NA	5.6	0	4.0	0
WT FCT	39	F	No	2.6	86.2	224.0	NA	27.5	6.4	0	0.1	0
WT FCT	20	F	NA	1.3	55.4	72.0	2.1	NA	3.6	4	3.4	0
WT FCT	46	F	Yes	2.8	58.6	164.0	NA	32.0	NA	0	<1	0
WT FCT	33	F	No	0.6	47.8	28.7	8.0	NA	NA	12	2.0	0
USP+ FCT	26	F	NA	1.4	235.7	330.0	NA	NA	3.0	NA	2.0	0

USP8+ FCT	45	F	NA	1.6	127.5	204.0	13.9	48.7	16.8	12	0.2	1
USP8+ FCT	37	F	No	0.9	53.0	47.7	4.7	11.3	1.6	12	0.9	0
USP8+ FCT	52	F	No	0.7	58.6	41.0	4.6	21.4	NA	12	0.5	0
USP8+ FCT	23	F	NA	1.2	46.9	56.3	6.3	24.3	44.2	12	2.0	4
USP8+ FCT	49	F	No	0.9	67.1	60.4	1.7	NA	1.9	12	1.5	0
USP8+ FCT	40	F	No	1.3	50.5	65.7	NA	NA	3.8	12	4.3	0
USP8+ FCT	40	F	No	0.8	110.0	88.0	1.1	4.3	2.4	1	3.5	1
USP8+ FCT	31	F	No	1.0	63.8	63.8	NA	NA	4.3	9	11.5	4
USP8+ FCT	31	F	No	1.1	90.9	100.0	NA	24.6	1.6	12	5.2	1
USP8+ FCT	26	F	No	0.4	162.5	65.0	NA	26.3	3.5	8	11.0	0

NFCT: non-functioning corticotroph tumor; FCT: functioning corticotroph tumor; *USP8*+: tumor with a *USP8* pathogenic variant; WT: wild type; ACTH: adrenocorticotrophic hormone; LNSC: late night salivary cortisol; ULN: upper limit of normal; UFC: urinary free cortisol; DST: dexamethasone suppression test; HPF: high power field; SST5: somatostatin receptor type 5; IRS: immunoreactive score; NA: not available.

Sup. Table 2. Correlations between gene expressions and demographic, biochemical, radiological and histopathological findings

	EGFR	*PCSK1*	*PCSK1N*	*POMC*	*TBX19*	*PAX6*
Age	*p*=0.847	**R=-0.335** **_p_=0.006**	*p*=0.341	**R=-0.398** **_p_<0.001**	*p*=0.205	**R=-0.266** **_p_=0.031**
Cortisol post DST	*p*=0.976	*p*=0.563	*p*=0.090	*p*=0.693	*p*=0.302	*p*=0.915
LNSC (xULN)	*p*=0.852	*p*=0.995	*p*=0.347	*p*=0.211	*p*=0.473	*p*=0.832
UFC (xULN)	*p*=0.448	*p*=0.110	*p*=0.579	*p*=0.821	**R=-0.686** **_p_=0.012**	*p*=0.448
ACTH	*p*=0.062	*p*=0.991	*p*=0.877	*p*=0.173	*p*=0.610	*p*=0.384
Largest tumor diameter	*p*=0.121	**R=-0.531** **_p_<0.001**	*p*=0.785	**R=-0.503** **_p_<0.001**	**R=-0.241** **_p_<0.001**	**R=-0.547** **_p_<0.001**
Secretion index	*p*=0.356	**R=+0.392** **_p_=0.024**	*p*=0.275	*p*=0.173	*p*=0.131	**R=+ 0.375** **_p_=0.031**
Ki-67	*p*=0.498	*p*=0.136	*p*=0.965	*p*=0.706	**R=-0.132** **_p_=0.029**	*p*=0.171
Mitoses	*p*=0.191	*p*=0.588	*p*=0.941	*p*=0.568	*p*=0.291	*p*=0.545
SST5 IRS	*p*=0.102	**R=0.552** **_p_<0.001**	*p*=0.924	**R=+ 0.343** **_p_=0.041**	**R=+0.393** **p=0.018**	**R=+0.567** **_p_<0.001**

NFCT: non-functioning corticotroph tumor; FCT: functioning corticotroph tumor; *USP8*+: tumor with a *USP8* pathogenic variant; WT: wild type; ACTH: adrenocorticotrophic hormone; LNSC: late night salivary cortisol; ULN: upper limit of normal; UFC: urinary free cortisol; DST: dexamethasone suppression test; HPF: high power field; SST5: somatostatin receptor type 5; IRS: immunoreactive score; NA: not available.
